# Bioinformatic analysis identifies potential biomarkers and therapeutic targets of septic-shock-associated acute kidney injury

**DOI:** 10.1186/s41065-021-00176-y

**Published:** 2021-04-16

**Authors:** Yun Tang, Xiaobo Yang, Huaqing Shu, Yuan Yu, Shangwen Pan, Jiqian Xu, You Shang

**Affiliations:** Department of Critical Care Medicine, Union Hospital, Tongji Medical College, Huazhong University of Science and Technology, No.1277, Jiefang Avenue, Wuhan, 430022 China

**Keywords:** Sepsis, Septic shock, Acute kidney injury, Bioinformatic analysis, Differentially expressed genes

## Abstract

**Background:**

Sepsis and septic shock are life-threatening diseases with high mortality rate in intensive care unit (ICU). Acute kidney injury (AKI) is a common complication of sepsis, and its occurrence is a poor prognostic sign to septic patients. We analyzed co-differentially expressed genes (co-DEGs) to explore relationships between septic shock and AKI and reveal potential biomarkers and therapeutic targets of septic-shock-associated AKI (SSAKI).

**Methods:**

Two gene expression datasets (GSE30718 and GSE57065) were downloaded from the Gene Expression Omnibus (GEO). The GSE57065 dataset included 28 septic shock patients and 25 healthy volunteers and blood samples were collected within 0.5, 24 and 48 h after shock. Specimens of GSE30718 were collected from 26 patients with AKI and 11 control patents. AKI-DEGs and septic-shock-DEGs were identified using the two datasets. Subsequently, Gene Ontology (GO) functional analysis, Kyoto Encyclopedia of Genes and Genomes (KEGG) pathway enrichment analysis, and protein-protein interaction (PPI) network analysis were performed to elucidate molecular mechanisms of DEGs. We also evaluated co-DEGs and corresponding predicted miRNAs involved in septic shock and AKI.

**Results:**

We identified 62 DEGs in AKI specimens and 888, 870, and 717 DEGs in septic shock blood samples within 0.5, 24 and 48 h, respectively. The hub genes of *EGF* and *OLFM4* may be involved in AKI and *QPCT*, *CKAP4*, *PRKCQ*, *PLAC8*, *PRC1*, *BCL9L*, *ATP11B*, *KLHL2*, *LDLRAP1*, *NDUFAF1*, *IFIT2*, *CSF1R*, *HGF*, *NRN1*, *GZMB*, and *STAT4* may be associated with septic shock. Besides, co-DEGs of *VMP1*, *SLPI*, *PTX3*, *TIMP1*, *OLFM4*, *LCN2*, and *S100A9* coupled with corresponding predicted miRNAs, especially miR-29b-3p, miR-152-3p, and miR-223-3p may be regarded as promising targets for the diagnosis and treatment of SSAKI in the future.

**Conclusions:**

Septic shock and AKI are related and *VMP1*, *SLPI*, *PTX3*, *TIMP1*, *OLFM4*, *LCN2*, and *S100A9* genes are significantly associated with novel biomarkers involved in the occurrence and development of SSAKI.

**Supplementary Information:**

The online version contains supplementary material available at 10.1186/s41065-021-00176-y.

## Background

Sepsis is a life-threatening disease which is defined as severe organ dysfunction results from a dysregulated innate immune response following infection. Septic shock is a subset of sepsis and is associated with severe cellular, metabolic, and circulatory abnormalities [1]. Currently, sepsis is one of the major causes of death in intensive care unit (ICU), with a mortality rate ranging from 20 to 50% of all cases [2].

Acute kidney injury (AKI) is a common complication of sepsis with an incidence between 47 to 61% [3, 4]. The morbidity of AKI in the context of septic patients is a poor prognostic sign and is correlated with higher mortality, increased length of ICU stay and hence considerable healthcare resources consumption [5]. Septic AKI carries a high mortality rate of up to 70% [6–9]. Due to its high morbidity and mortality in critically ill patients, it is of great importance to identify those septic patients at highest risk of developing AKI.

Diagnosis for septic AKI has been and is still dependent on serum creatinine and urine output [10]. However, the two functional markers have serious limitations that may lead to late or even missed diagnosis. Serum creatinine concentrations might not change until 48–72 h after the initial insult to the kidney, and tend to rise when about 50% of kidney function has already been lost [11]. Moreover, renal injury can exist with no change or only slightly increase in creatinine due to renal reserve or tubular secretion of creatinine [12]. In addition, an oliguria or an increase in creatinine may be also caused by renal hypoperfusion due to prerenal factors although kidney function is not impaired [13]. Consequently, the effectiveness of therapy for septic AKI is largely limited by the diagnosis that based upon changes in serum creatinine and urine output. In order to identify and develop robust diagnostic biomarkers and therapeutic strategies for septic AKI, a better understanding of the mechanisms leading to renal damage as well as recovery in septic patients is essential.

In this study, two datasets were downloaded and analyzed from the Gene Expression Omnibus (GEO) to identified genes that are co-differentially expressed (co-DEGs) in septic shock and AKI. Then, we elucidated molecular mechanisms of septic-shock-related DEGs and AKI-related DEGs through functional and pathway analyses and protein-protein interaction (PPI) network analysis. Finally, we predicted microRNAs (miRNAs) that may be involved in the process of septic shock patients prone to AKI.

## Materials and methods

### Microarray data

Two gene expression datasets (GSE30718 [14] and GSE57065 [15]) were downloaded from the GEO (http://www.ncbi.nlm.nih.gov/geo/) [16] and the platform used for both expression profiling arrays was the GPL570 [HG-U133_Plus_2] Affymetrix Human Genome U133 Plus 2.0 Array (Affymetrix, Santa Clara, CA, USA). The two datasets have included all necessary information and no samples had to be taken on site.

The GSE57065 dataset included 28 septic shock patients and 25 healthy volunteers and blood samples were collected within 0.5, 24 and 48 h after shock. Patients aged < 18, having one or more severe comorbidities, or receiving immunosuppressive therapy were excluded. The diagnosis of septic shock used the ACCP/SCCM criteria. The onset of the septic shock was defined as the beginning of vasopressor therapy. Specimens of GSE30718 were collected from 26 patients with AKI and 11 control patents. The AKI cohort consisted of 28 biopsies from 26 kidney transplants with acute injury, with a mean estimated glomerular filtration rate (eGFR) at biopsy of 26 ml/min. Controls consisted of 11 pristine protocol biopsies from stable kidney transplants with no histologic abnormalities, with a mean eGFR at biopsy of 51.2 ml/min. All RNA information of the selected samples was downloaded for further analysis. Ethical approval was not necessary because our study is bioinformatic analysis.

### Identification of DEGs

The original expression matrix was processed by R software. The “limma” package [17] was utilized to screen out DEGs. Adjusted *P*-value < 0.05 and |Log fold-change| > 1 were used for filtering septic-shock-DEGs and AKI-DEGs. DEGs from the two datasets were screened for subsequent Gene Ontology (GO), Kyoto Encyclopedia of Genes and Genomes (KEGG) pathway enrichment analyses, and PPI network analysis. In addition, co-DEGs for AKI and septic shock were calculated and made on Venn diagram.

### PPI network construction and hub gene identification

PPI networks of AKI- and septic-shock-DEGs were constructed using the Search Tool for the Retrieval of Interacting Genes (STRING; http://string-db.org/). Interaction with a combined score > 0.4 of AKI-DEGs and > 0.9 of septic-shock-DEGs were set as the cut-off points. Subsequently, Cytoscape software was used to construct and visualize molecular interaction networks [18]. The hub genes in the PPI networks were selected using the plug-in Molecular Complex Detection (MCODE) of Cytoscape [19].

### GO and KEGG pathway enrichment analyses of DEGs

GO function analysis (cellular component [CC], biological process [BP], and molecular function [MF]) is a powerful bioinformatics tool to classify gene expression and its properties [20]. KEGG pathway analysis was used to find out which cell pathways might be involved in the changes in DEGs [21]. GO and KEGG pathway enrichment analyses of AKI- and septic-shock-DEGs were performed using the R package “clusterProfiler” [22]. A *P* < 0.05 was considered statistically significant.

### Associations between co-DEGs and kidney or infectious diseases

The comparative toxicogenomics database (CTD; http://ctdbase.org/) is a public resource that describes interactions of chemical-gene/protein, chemical-disease, and gene/protein-disease, providing information about interactions between environmental chemicals and gene products and their relationships to diseases [23]. Thus, we used these data to analyze and identify the associations between co-DEGs and kidney or infectious diseases.

### Functional enrichment in co-DEGs

The AmiGO database (v2.0; http://amigo.geneontology.org/amigo/) is a web-based application for querying, browsing, visualizing, and downloading the Gene Ontology and annotations [24]. We used the AmiGO database to comfirm GO term enrichment for identified co-DEGs involved in AKI and septic shock.

### GO and KEGG pathway enrichment among predicted miRNAs and co-DEGs

We used the STARBASE Database ENCORI (http://starbase.sysu.edu.cn/) to predict potential microRNAs that may regulate co-DEGs [25]. Six prediction programs (PITA, miRmap, miRanda, microT, PicTar, and TargetScan) were utilized to determine candidate miRNAs of each co-DEG. Subsequently, we applied computational tool Diana-miRPath (v3.0; http://www.microrna.gr/miRPathv3) to assess interactions among miRNAs previously identified and co-DEGs [26].

## Results

### Identification of DEGs

**Fig. 1:**
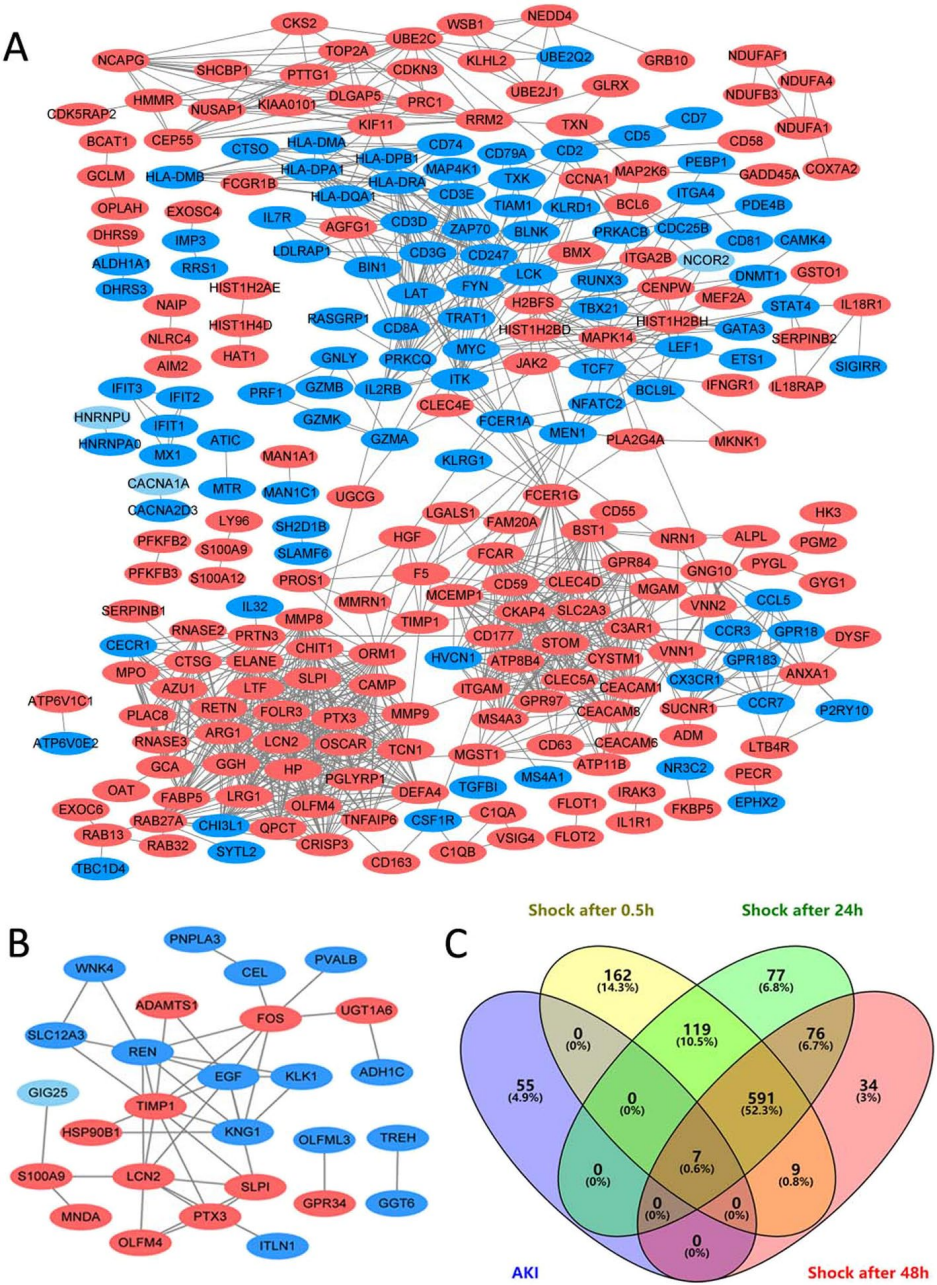
PPI network and Venn diagrams. **a**, **b** The PPI networks of septic-shock-DEGs and AKI-DEGs constructed using Cytoscape. Red represents upregulated genes, and blue represents downregulated genes. **c** Venn diagram of DEGs related to AKI and within 0.5, 24, and 48 h after septic shock, respectively. Seven co-expressed genes, including *VMP1*, *SLPI*, *PTX3*, *TIMP1*, *OLFM4*, *LCN2*, and *S100A9*, are identified

In total, 62 DEGs were extracted from the AKI samples, among which 22 genes were up-regulated and 40 genes were down-regulated (Additional file 1). In addition, we identified 888, 870, and 717 DEGs within 0.5, 24 and 48 h after septic shock, respectively (Additional file 2, Additional file 3 and Additional file 4). Here, 598 co-expressed DEGs in the three time points mentioned above were confirmed as septic-shock-DEGs. The Venn diagram were presented in Fig. 1c.

### PPI network construction and hub gene identification

We identified 27 nodes and 266 nodes from PPI network of AKI- and septic-shock-DEGs, respectively (Fig. 1a, b). Two hub nodes, involved in epidermal growth factor (*EGF*) and olfactomedin 4 (*OLFM4*), are considering as hub genes related to AKI. A total of 16 genes, including glutaminyl-peptide cyclotransferase (*QPCT*), cytoskeleton-associated protein 4 (*CKAP4*), protein kinase C, theta (*PRKCQ*), placenta-specific 8 (*PLAC8*), protein regulator of cytokinesis 1 (*PRC1*), B-cell CLL/lymphoma 9-like (*BCL9L*), ATPase, class VI, type 11B (*ATP11B*), kelch-like family member 2 (*KLHL2*), low density lipoprotein receptor adaptor protein 1 (*LDLRAP1*), NADH dehydrogenase complex I, assembly factor 1 (*NDUFAF1*), interferon-induced protein with tetratricopeptide repeats 2 (*IFIT2*), colony stimulating factor 1 receptor (*CSF1R*), hepatocyte growth factor (*HGF*), neuritin 1 (*NRN1*), granzyme B (*GZMB*), and signal transducer and activator of transcription 4 (*STAT4*), are identified as hub genes associated with septic shock.

### GO and KEGG pathway enrichment analyses of DEGs

**Fig. 2:**
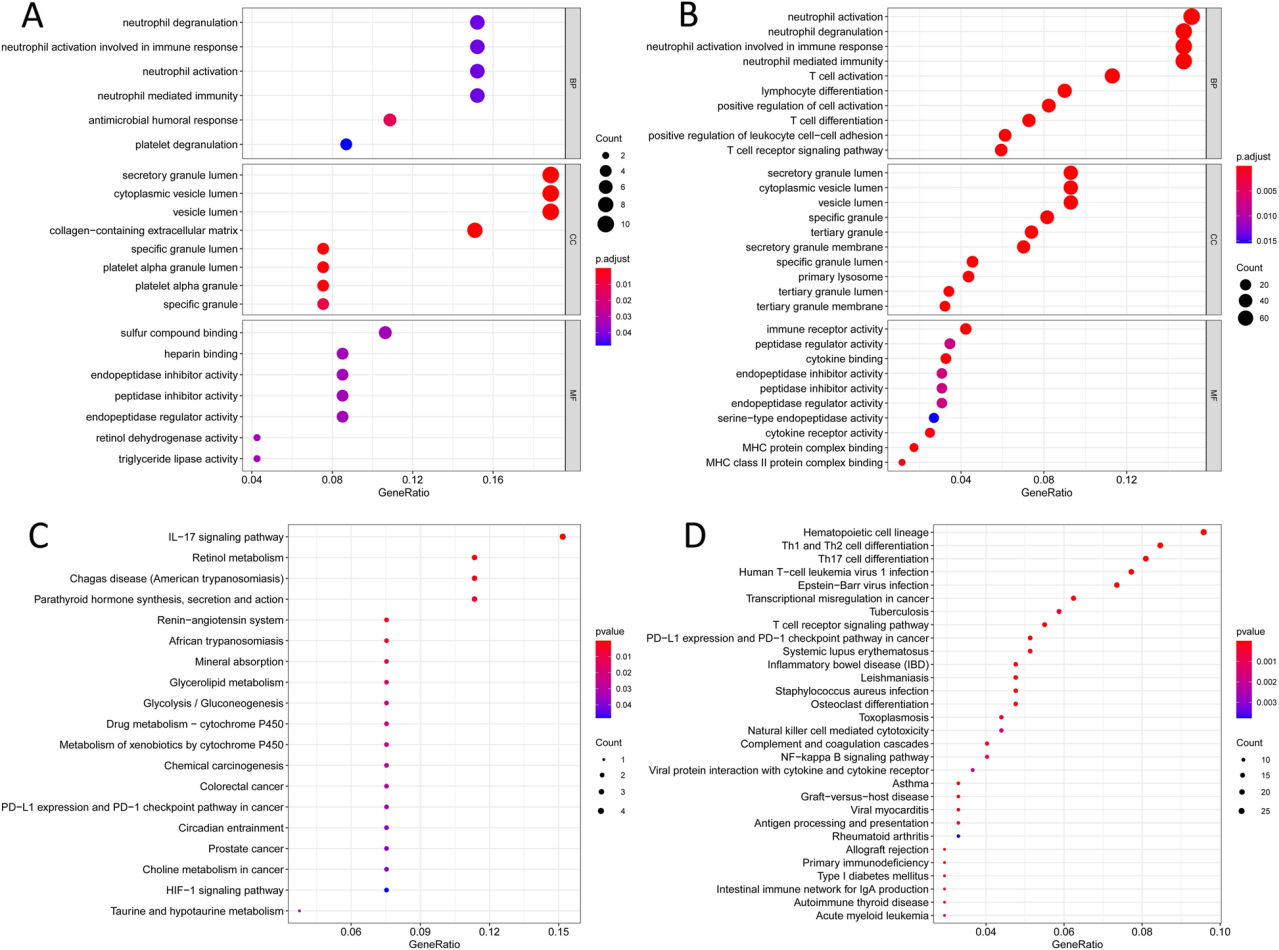
GO terms and KEGG pathway enrichment. **a**, **b** GO categories of AKI- and septic-shock-DEGs, respectively. **c**, **d** KEGG pathway enrichment of AKI- and septic-shock-DEGs, respectively

With respect to AKI-DEGs, the GO terms related BP were mainly enriched in neutrophil degranulation/activation, neutrophil mediated immunity, and antimicrobial humoral response. CC were primarily associated with vesicle lumen, secretory granule lumen, and extracellular matrix. MF were mainly involved in sulfur compound binding, heparin binding, and peptidase inhibitor activity. As for septic-shock-DEGs, the BP terms of neutrophil activation/degranulation, neutrophil mediated immunity, and T cell activation were significantly enriched. There is significant correlation in vesicle lumen, secretory granule lumen, and specific granule in relation to CC. Similarly, MF were mainly enriched in immune receptor activity, peptidase regulator activity, and cytokine binding (Fig. 2).

KEGG pathway enrichment analysis of AKI-DEGs were mainly enriched in IL − 17 signaling pathway, retinol metabolism, chagas disease, and parathyroid hormone synthesis. KEGG terms included hematopoietic cell lineage, Th1 and Th2 cell differentiation, Th17 cell differentiation, and human T-cell leukemia virus 1 infection were enriched in septic-shock-DEGs (Fig. 2).

### Functional enrichment in co-DEGs

Seven co-expressed DEGs, including vacuole membrane protein 1 (*VMP1*), secretory leukocyte peptidase inhibitor (*SLPI*), pentraxin 3 (*PTX3*), TIMP metallopeptidase inhibitor 1 (*TIMP1*), olfactomedin 4 (*OLFM4*), lipocalin 2 (*LCN2*), S100 calcium binding protein A9 (*S100A9*), were observed. The CTD database revealed that co-DEGs targeted various kidney and infectious diseases (Table 1). The AmiGO database was used to recognize GO consortium of co-DEGs and these data appear in Table 2.

**Table 1:** Relationship to infectious and kidney diseases related to co-DEGs based on the CTD database

Genes	Disease name	Disease ID	Inference score	Reference count
VMP1	Proteus Infections	MESH:D011512	7.77	2
	Acinetobacter Infections	MESH:D000151	4	1
	Bacterial Infections	MESH:D001424	3.79	1
	Kidney Diseases	MESH:D007674	74.98	274
	Acute Kidney Injury	MESH:D058186	57.3	164
	Kidney Tubular Necrosis, Acute	MESH:D007683	37.19	38
SLPI	Bacterial Infections	MESH:D001424	14.92	8
	Pneumonia, Pneumocystis	MESH:D011020	12.12	4
	Staphylococcal Infections	MESH:D013203	11.08	31
	Kidney Diseases	MESH:D007674	140.73	445
	Acute Kidney Injury	MESH:D058186	81.18	300
	Proteinuria	MESH:D011507	72.69	160
PTX3	Bacterial Infections	MESH:D001424	10.56	4
	Helicobacter Infections	MESH:D016481	9.69	3
	Pseudomonas Infections	MESH:D011552	6.47	2
	Kidney Diseases	MESH:D007674	107.5	348
	Acute Kidney Injury	MESH:D058186	88.8	205
	Proteinuria	MESH:D011507	83.53	150
TIMP1	Bacterial Infections	MESH:D001424	38.56	11
	IMMUNE SUPPRESSION	OMIM:146850	25.12	4
	Staphylococcal Infections	MESH:D013203	24.54	114
	Glomerulonephritis, IGA^a^	MESH:D005922	11.38	6
	Kidney Diseases	MESH:D007674	417.32	804
	Acute Kidney Injury	MESH:D058186	305.13	549
	Proteinuria	MESH:D011507	245.56	439
OLFM4	IMMUNE SUPPRESSION	OMIM:146850	4.94	1
	Hidradenitis Suppurativa	MESH:D017497	3.98	1
	Meningitis, Pneumococcal	MESH:D008586	3.65	1
	Kidney Diseases	MESH:D007674	52.19	67
	Acute Kidney Injury	MESH:D058186	33.22	21
	Proteinuria	MESH:D011507	31.39	24
LCN2	Meningitis, Cryptococcal	MESH:D016919	32.23	13
	Mycoses	MESH:D009181	30.46	30
	Candidiasis, Vulvovaginal	MESH:D002181	22.19	5
	Kidney Diseases^a^	MESH:D007674	328.81	776
	Acute Kidney Injury^a^	MESH:D058186	227.05	523
	Proteinuria	MESH:D011507	177.1	385
	Renal Insufficiency	MESH:D051437	140.84	170
S100A9	Bacterial Infections	MESH:D001424	22.04	8
	Staphylococcal Infections	MESH:D013203	18.13	15
	IMMUNE SUPPRESSION	OMIM:146850	17.92	4
	Kidney Diseases	MESH:D007674	274.34	537
	Acute Kidney Injury	MESH:D058186	214.94	463
	Renal Insufficiency	MESH:D051437	146.23	153

^a^Direct evidence of marker or mechanism in this disease

**Table 2:** The GO terms enrichment for the co-DEGs of septic shock and AKI

Gene/product	GO class (direct)	Evidence	Reference
VMP1	protein binding	IPI	PMID:17724469
	endoplasmic reticulum	IDA	GO_REF:0000052
	autophagosome assembly	IMP	PMID:30093494
SLPI	protein binding	IPI	PMID:12526812
	antibacterial humoral response	ISS	GO_REF:0000024
	extracellular space	IDA	PMID:18714013
	collagen-containing extracellular matrix	HDA	PMID:28675934
	innate immune response	ISS	GO_REF:0000024
	modulation of process of other organism	IDA	PMID:2467900
	negative regulation of endopeptidase activity	IEA	GO_REF:0000108
	serine-type endopeptidase inhibitor activity	IDA	PMID:3462719
PTX3	protein binding	IPI	PMID:19050261
	complement component C1q complex binding	IDA	PMID:23544079
	extracellular region	TAS	Reactome:R-HSA-6798745
	extracellular space	IDA	PMID:23544079
	innate immune response	IDA	PMID:23544079
TIMP1	protein binding	IPI	PMID:16917503
	extracellular region	TAS	Reactome:R-HSA-1602454
	metalloendopeptidase inhibitor activity	IDA	PMID:12714508
	basement membrane	IEA	GO_REF:0000107
	cytokine-mediated signaling pathway	TAS	Reactome:R-HSA-6783783
	extracellular space	IDA	PMID:3903517
	negative regulation of endopeptidase activity	IDA	PMID:3903517
	negative regulation of membrane protein ectodomain proteolysis	IDA	PMID:12714508
OLFM4	protein binding	IPI	PMID:20534456
	extracellular exosome	HDA	PMID:19056867
	extracellular space	HDA	PMID:16502470
	extracellular region	TAS	Reactome:R-HSA-6798745
	plasma membrane	IDA	GO_REF:0000052
LCN2	protein binding	IPI	PMID:32296183
	extracellular region	ISS	GO_REF:0000024
	extracellular space	HDA	PMID:16502470
	extracellular exosome	HDA	PMID:19056867
	iron ion binding	ISS	GO_REF:0000024
S100A9	protein binding	IPI	PMID:17620599
	extracellular region	TAS	PMID:22489132
	collagen-containing extracellular matrix	HDA	PMID:23979707
	extracellular space	HDA	PMID:16502470
	cytosol	IDA	GO_REF:0000052
	extracellular exosome	HDA	PMID:19056867
	antimicrobial humoral immune response mediated by antimicrobial peptide	IDA	PMID:12874352
	calcium ion binding	TAS	PMID:22489132
	cytoplasm	IDA	PMID:12874352
	leukocyte migration involved in inflammatory response	IDA	PMID:12626582
	neutrophil aggregation	IDA	PMID:12626582
	neutrophil chemotaxis	IDA	PMID:12626582
	nucleus	HDA	PMID:21630459

*IPI* physical interaction evidence used in manual assertion, *IDA* direct assay evidence used in manual assertion, *ISS* sequence similarity evidence used in manual assertion, *IEA* evidence used in automatic assertion, *TAS* traceable author statement used in manual assertion, *IMP* mutant phenotype evidence used in manual assertion, *HAD* high throughput direct assay evidence used in manual assertion

### GO and KEGG pathway enrichment among predicted miRNAs and co-DEGs

The miRNAs targeting each co-DEG were selected if they were predicted by two or more prediction programs. The analyses of GO and KEGG pathway enrichment between predicted miRNAs and co-DEGs were shown in Table 3. These analyses made us clearness the molecular mechanisms how predicted miRNAs influence septic-shock-associated AKI (SSAKI).

**Table 3:** GO and KEGG pathways enrichment among predicted miRNAs and co-DEGs

Genes	Predicted miRNAs	Category		***P*** value
SLPI	hsa-miR-370-3p	KEGG pathway	NA	
	hsa-miR-3679-5p	GO terms	NA	
	hsa-miR-520a-5p			
TIMP1	hsa-miR-22-3p	KEGG pathway	HIF-1 signaling pathway	9e-05
	hsa-miR-4739	GO terms	regulation of integrin-mediated signaling pathway	0.002
	hsa-miR-1321		negative regulation of metalloenzyme activity	0.002
	hsa-miR-4756-5p		negative regulation of trophoblast cell migration	0.002
			negative regulation of membrane protein ectodomain proteolysis	0.002
			cell activation	0.005
			metalloendopeptidase inhibitor activity	0.005
			platelet alpha granule lumen	0.005
			platelet degranulation	0.008
			extracellular matrix disassembly	0.012
			response to peptide hormone	0.015
PTX3	hsa-miR-29a-3p	KEGG pathway	NA	
	hsa-miR-29b-3p	GO terms	NA	
	hsa-miR-29c-3p			
	hsa-miR-101-3p			
	hsa-miR-144-3p			
VMP1	hsa-miR-19a-3p	KEGG pathway	MicroRNAs in cancer	3e-24
	hsa-miR-19b-3p		Proteoglycans in cancer	0.010
	hsa-miR-148a-3p	GO terms	autophagic vacuole membrane	0.008
	hsa-miR-152-3p		pre-autophagosomal structure	0.008
	miR-223-3p		cell junction assembly	0.008
			regulation of autophagy	0.008
			embryo implantation	0.009
			cell junction organization	0.014
			autophagy	0.017
			single organismal cell-cell adhesion	0.017

## Discussion

The mortality of AKI induced by sepsis and septic shock has remained high in recent years. Early diagnosis, appropriate classification and timely treatments in the initial periods of septic AKI play a crucial role in reducing mortality. Bioinformatic analyses enable us to understand the molecular mechanisms of disease occurrence and development, providing a novel and effective way to identify potential diagnostic biomarkers and therapeutic targets in preventing and treating septic AKI. In the present study, seven co-DEGs of septic shock and AKI, including *VMP1*, *SLPI*, *PTX3*, *TIMP1*, *OLFM4*, *LCN2* and *S100A9*, were identified.

*TIMP1* belongs to the *TIMP* gene family and the proteins encoded by this gene family are known to inhibit the matrix metalloproteinases (MMPs) activity and regulate the balance of matrix remodeling during degradation of the extracellular matrix [27]. Bojic et al. compared 53 patients with sepsis divided into sepsis-associated acute kidney injury (SAAKI) group and non-SAAKI group to 50 controls without sepsis [28]. They found that the patients with SAAKI had higher serum *TIMP1* expression compared to septic patients without SAAKI and the control group. Their findings suggested *TIMP1* could serve as potential diagnostic biomarker of sepsis with AKI. *OLFM4* gene encodes a highly glycosylated protein which contains the olfactomedin domain [29]. The encoded protein has been proved to regulate various cellular functions such as cell growth, apoptosis, differentiation and proliferation [30]. Stark et al. reported that wild type mice had a significant increase in serum creatinine and renal cell apoptosis at 24 h after cecal slurry injection while it was not observed in *OLFM4* null mice. This revealed that *OLFM4* expression may be involved with kidney injury in sepsis [31]. A retrospective cohort study demonstrated that upregulation of *OLFM4* in peripheral blood was associated with SSAKI via transcriptome analysis [32]. These findings indicated that the *OLFM4* gene may be a potential biomarker and prognostic indicator for SSAKI. *LCN2*, also known as neutrophil gelatinase-associated lipocalin (*NGAL*), is a member of the lipocalin superfamily. The protein encoded by *LCN2* is involved in the transport of small hydrophobic molecules [33]. Wang et al. reported that the expression of *NGAL* was much higher in AKI than in non-AKI septic patients and the AUC of *NGAL* expression for predicting AKI in septic patients was higher than that of procalcitonin (PCT) [34]. The present study proved that *NGAL* may be an emerging diagnostic biomarker for AKI in septic patients. The protein encoded by *S100A9* is a member of the S100 family of calcium-binding proteins that promotes and exacerbates the inflammatory response [35]. Study have reported that patients with higher serum *S100A9* expression tended to suffer from more severe sepsis-related organ dysfunction [36]. Leeds et al. observed that expression of *S100A9* was drastically increased in iMCD3 (inner medullary collecting duct cell line) cells from sepsis-induced AKI model exposed to lipopolysaccharide (LPS) serum compared to control group exposed to phosphate-buffered saline (PBS) serum [37]. The studies suggested *S100A9* could be potential diagnostic biomarker of sepsis-induced AKI. *VMP1*, which was first observed in pancreatitis, encoding a multispanning membrane protein in the endoplasmic reticulum, participating in the process of autophagy [38]. *SLPI* gene encodes a protease inhibitor and regulator of innate and adaptive immunity [39]. It is revealed that the expressed *SLPI* was elevated in patients with sepsis and the level of elevation was associated with the severity of organ dysfunction [40]. *PTX3* is the prototype of the long pentraxin subfamily, playing an important role in regulating humoral innate immune response and participating in innate resistance to pathogens [41]. However, the role of *VMP1*, *SLPI*, *PTX3* in the development of SAAKI remains unclear. Further studies will be required to identify the relationship between these genes and SSAKI.

miRNA comprise a class of small noncoding RNAs, regulating protein expression through degeneration or inhibition translation when binding to mRNA [42]. Recent studies have shown that miRNAs were closely involved with the occurrence, development, and prognosis of septic AKI [43, 44]. Our study identified several miRNAs targeting each co-DEG involved with sepsis and AKI. Xu et al. found that miR-29b-3p were implicated in PI3K-Akt signaling pathway, suggesting it might be associated with the pathogenesis of sepsis-induced AKI [45]. Ma et al. observed that the expression of miR-152-3p was upregulated in the serum of septic AKI patients and positively related to serum creatinine, urea nitrogen, interleukin 1β and tumor necrosis factor α, suggesting the extent of miR-152-3p elevation correlated to the severity of kidney injury and inflammatory response [46]. Tan et al. reported that DLX6-AS1 mediated HK-2 cell pyroptosis in LPS-induced AKI via repress miR-223-3p expression in HK-2 cells, showing enhanced miR-223-3p expression may serve a new strategy for more effective control of septic AKI [47]. These findings shown that miR-29b-3p, miR-152-3p, and miR-223-3p may be potential targets for the treatment of SSAKI. However, the effect and possible mechanism of other predicted miRNAs in SSAKI is unclear, and further exploration will be required to reveal the correlation between the miRNAs and pathogenesis of SSAKI to provide theoretical support for its clinical treatment.

The limitation of our study is that these identified biomarkers are currently limited to the theoretical level. Further experimental studies and clinical trials should be carried out to obtain accurate verification and validate our results.

## Conclusions

The hub genes of *EGF* and *OLFM4* may be involved in the occurrence and progression of AKI and *QPCT*, *CKAP4*, *PRKCQ*, *PLAC8*, *PRC1*, *BCL9L*, *ATP11B*, *KLHL2*, *LDLRAP1*, *NDUFAF1*, *IFIT2*, *CSF1R*, *HGF*, *NRN1*, *GZMB*, and *STAT4* may be associated with septic shock. Besides, co-DEGs of *VMP1*, *SLPI*, *PTX3*, *TIMP1*, *OLFM4*, *LCN2*, and *S100A9* were identified to link AKI and septic shock. Finally, predicted miRNAs for each co-DEGs may be regarded as potential biomarkers or therapeutic targets for SSAKI, especially miR-29b-3p, miR-152-3p, and miR-223-3p.

## Supplementary Information

**Additional file 1.** Differentially expressed genes involved in AKI samples.

**Additional file 2.** Differentially expressed genes involved in septic shock samples within 0.5 hours.

**Additional file 3.** Differentially expressed genes involved in septic shock samples within 24 hours.

**Additional file 4.** Differentially expressed genes involved in septic shock samples within 48 hours.

## Data Availability

All data generated or analysed during this study are included in this published article [and its Supplementary information files].

## Abbreviations

AKI: Acute kidney injury
BP: Biological process
CC: Cellular component
CTD: Comparative toxicogenomics database
DEGs: Differentially expressed genes
eGFR: Estimated glomerular filtration rate
GEO: Gene expression omnibus
GO: Gene Ontology
iMCD3: Inner medullary collecting duct cell line
ICU: Intensive care unit
KEGG: Kyoto Encyclopedia of Genes and Genomes
LPS: Lipopolysaccharide
miRNAs: microRNAs
MCODE: Molecular Complex Detection
MF: Molecular function
MMPs: Matrix metalloproteinases
PPI: Protein-protein interaction
PCT: Procalcitonin
PBS: Phosphate-buffered saline
STRING: Search Tool for the Retrieval of Interacting Genes
SSAKI: Septic-shock-associated acute kidney injury
SAAKI: Sepsis-associated acute kidney injury

## References

[1] Singer M, Deutschman CS, Seymour CW, Shankar-Hari M, Annane D, Bauer M, Bellomo R, Bernard GR, Chiche J, Coopersmith CM (2016). The third international consensus definitions for sepsis and septic shock (Sepsis-3). JAMA.

[2] Huet O, Chin-Dusting JP (2014). Septic shock: desperately seeking treatment. Clin Sci.

[3] Plataki M, Kashani K, Cabello-Garza J, Maldonado F, Kashyap R, Kor DJ, Gajic O, Cartin-Ceba R (2011). Predictors of acute kidney injury in septic shock patients: an observational cohort study. Clin J Am Soc Nephrol.

[4] Uchino S, Kellum JA, Bellomo R, Doig GS, Morimatsu H, Morgera S, Schetz M, Tan I, Bouman C, Macedo E (2005). Acute renal failure in critically ill patients: a multinational, multicenter study. JAMA..

[5] Zarjou A, Agarwal A (2011). Sepsis and acute kidney injury. J Am Soc Nephrol.

[6] Bagshaw SM, George C, Bellomo R (2008). Early acute kidney injury and sepsis: a multicentre evaluation. Crit Care.

[7] Bagshaw SM, Uchino S, Bellomo R, Morimatsu H, Morgera S, Schetz M, Tan I, Bouman C, Macedo E, Gibney N (2007). Septic acute kidney injury in critically ill patients: clinical characteristics and outcomes. Clin J Am Soc Nephrol.

[8] Schrier RW, Wang W (2004). Acute renal failure and sepsis. N Engl J Med.

[9] Neveu H, Kleinknecht D, Brivet F, Loirat P, Landais P (1996). Prognostic factors in acute renal failure due to sepsis. Results of a prospective multicentre study. The French study group on acute renal failure. Nephrol Dial Transplant.

[10] Thomas ME, Blaine C, Dawnay A, Devonald MA, Ftouh S, Laing C, Latchem S, Lewington A, Milford DV, Ostermann M (2015). The definition of acute kidney injury and its use in practice. Kidney Int.

[11] Mishra J, Dent C, Tarabishi R, Mitsnefes MM, Ma Q, Kelly C, Ruff SM, Zahedi K, Shao M, Bean J (2005). Neutrophil gelatinase-associated lipocalin (NGAL) as a biomarker for acute renal injury after cardiac surgery. Lancet..

[12] Herrera J, Rodríguez-Iturbe B (1998). Stimulation of tubular secretion of creatinine in health and in conditions associated with reduced nephron mass. Evidence for a tubular functional reserve. Nephrol Dial Transplant.

[13] Ichai C, Vinsonneau C, Souweine B, Armando F, Canet E, Clec'H C, Constantin JM, Darmon M, Duranteau J, Gaillot T (2016). Acute kidney injury in the perioperative period and in intensive care units (excluding renal replacement therapies). Ann Intensive Care.

[14] Famulski KS, de Freitas DG, Kreepala C, Chang J, Sellares J, Sis B, Einecke G, Mengel M, Reeve J, Halloran PF (2012). Molecular phenotypes of acute kidney injury in kidney transplants. J Am Soc Nephrol.

[15] Cazalis MA, Lepape A, Venet F, Frager F, Mougin B, Vallin H, Paye M, Pachot A, Monneret G (2014). Early and dynamic changes in gene expression in septic shock patients: a genome-wide approach. Intensive Care Med Exp.

[16] Barrett T, Wilhite SE, Ledoux P, Evangelista C, Kim IF, Tomashevsky M, Marshall KA, Phillippy KH, Sherman PM, Holko M (2013). NCBI GEO: archive for functional genomics data sets--update. Nucleic Acids Res.

[17] Ritchie ME, Phipson B, Wu D, Hu Y, Law CW, Shi W, Smyth GK (2015). Limma powers differential expression analyses for RNA-sequencing and microarray studies. Nucleic Acids Res.

[18] Shannon P, Markiel A, Ozier O, Baliga NS, Wang JT, Ramage D, Amin N, Schwikowski B, Ideker T (2003). Cytoscape: a software environment for integrated models of biomolecular interaction networks. Genome Res.

[19] Bandettini WP, Kellman P, Mancini C, Booker OJ, Vasu S, Leung SW, Wilson JR, Shanbhag SM, Chen MY, Arai AE (2012). MultiContrast delayed enhancement (MCODE) improves detection of subendocardial myocardial infarction by late gadolinium enhancement cardiovascular magnetic resonance: a clinical validation study. J Cardiovasc Magn Reson.

[20] Ashburner M, Ball CA, Blake JA, Botstein D, Butler H, Cherry JM, Davis AP, Dolinski K, Dwight SS, Eppig JT (2000). Gene ontology: tool for the unification of biology. The gene ontology consortium. Nat Genet.

[21] Altermann E, Klaenhammer TR (2005). PathwayVoyager: pathway mapping using the Kyoto encyclopedia of genes and genomes (KEGG) database. BMC Genomics.

[22] Yu G, Wang LG, Han Y, He QY (2012). clusterProfiler: an R package for comparing biological themes among gene clusters. OMICS..

[23] Davis AP, Grondin CJ, Johnson RJ, Sciaky D, King BL, McMorran R, Wiegers J, Wiegers TC, Mattingly CJ (2017). The comparative toxicogenomics database: update 2017. Nucleic Acids Res.

[24] Carbon S, Ireland A, Mungall CJ, Shu S, Marshall B, Lewis S (2009). AmiGO: online access to ontology and annotation data. Bioinformatics..

[25] Li JH, Liu S, Zhou H, Qu LH, Yang JH (2014). starBase v2.0: decoding miRNA-ceRNA, miRNA-ncRNA and protein-RNA interaction networks from large-scale CLIP-Seq data. Nucleic Acids Res.

[26] Paraskevopoulou MD, Georgakilas G, Kostoulas N, Vlachos IS, Vergoulis T, Reczko M, Filippidis C, Dalamagas T, Hatzigeorgiou AG (2013). DIANA-microT web server v5.0: service integration into miRNA functional analysis workflows. Nucleic Acids Res.

[27] Batra J, Robinson J, Soares AS, Fields AP, Radisky DC, Radisky ES (2012). Matrix metalloproteinase-10 (MMP-10) interaction with tissue inhibitors of metalloproteinases TIMP-1 and TIMP-2: binding studies and crystal structure. J Biol Chem.

[28] Bojic S, Kotur-Stevuljevic J, Kalezic N, Stevanovic P, Jelic-Ivanovic Z, Bilanovic D, Memon L, Damnjanovic M, Kalaba Z, Simic-Ogrizovic S (2015). Diagnostic value of matrix metalloproteinase-9 and tissue inhibitor of matrix metalloproteinase-1 in sepsis-associated acute kidney injury. Tohoku J Exp Med.

[29] Zhang J, Liu WL, Tang DC, Chen L, Wang M, Pack SD, Zhuang Z, Rodgers GP (2002). Identification and characterization of a novel member of olfactomedin-related protein family, hGC-1, expressed during myeloid lineage development. Gene..

[30] Li J, Liu C, Li D, Wan M, Zhang H, Zheng X, Jie X, Zhang P, Li J, Hou H (2019). OLFM4 inhibits epithelial-mesenchymal transition and metastatic potential of cervical cancer cells. Oncol Res.

[31] Stark JE, Opoka AM, Mallela J, Devarajan P, Ma Q, Levinsky NC, Stringer KF, Wong HR, Alder MN (2020). Juvenile OLFM4-null mice are protected from sepsis. Am J Physiol Ren Physiol.

[32] Basu RK, Standage SW, Cvijanovich NZ, Allen GL, Thomas NJ, Freishtat RJ, Anas N, Meyer K, Checchia PA, Lin R (2011). Identification of candidate serum biomarkers for severe septic shock-associated kidney injury via microarray. Crit Care.

[33] Goetz DH, Holmes MA, Borregaard N, Bluhm ME, Raymond KN, Strong RK (2002). The neutrophil lipocalin NGAL is a bacteriostatic agent that interferes with siderophore-mediated iron acquisition. Mol Cell.

[34] Wang M, Zhang Q, Zhao X, Dong G, Li C (2014). Diagnostic and prognostic value of neutrophil gelatinase-associated lipocalin, matrix metalloproteinase-9, and tissue inhibitor of matrix metalloproteinases-1 for sepsis in the emergency department: an observational study. Crit Care.

[35] Koy M, Hambruch N, Hussen J, Pfarrer C, Seyfert HM, Schuberth HJ (2013). Recombinant bovine S100A8 and A9 enhance IL-1β secretion of interferon-gamma primed monocytes. Vet Immunol Immunopathol.

[36] Chen L, Long X, Xu Q, Tan J, Wang G, Cao Y, Wei J, Luo H, Zhu H, Huang L (2020). Elevated serum levels of S100A8/A9 and HMGB1 at hospital admission are correlated with inferior clinical outcomes in COVID-19 patients. Cell Mol Immunol.

[37] Leeds J, Scindia Y, Loi V, Wlazlo E, Ghias E, Cechova S, Portilla D, Ledesma J, Swaminathan S (2020). Protective role of DJ-1 in endotoxin-induced acute kidney injury. Am J Physiol Ren Physiol.

[38] Dusetti NJ, Jiang Y, Vaccaro MI, Tomasini R, Azizi SA, Calvo EL, Ropolo A, Fiedler F, Mallo GV, Dagorn JC (2002). Cloning and expression of the rat vacuole membrane protein 1 (VMP1), a new gene activated in pancreas with acute pancreatitis, which promotes vacuole formation. Biochem Biophys Res Commun.

[39] Thompson RC, Ohlsson K (1986). Isolation, properties, and complete amino acid sequence of human secretory leukocyte protease inhibitor, a potent inhibitor of leukocyte elastase. Proc Natl Acad Sci U S A.

[40] Grobmyer SR, Barie PS, Nathan CF, Fuortes M, Lin E, Lowry SF, Wright CD, Weyant MJ, Hydo L, Reeves F (2000). Secretory leukocyte protease inhibitor, an inhibitor of neutrophil activation, is elevated in serum in human sepsis and experimental endotoxemia. Crit Care Med.

[41] Bottazzi B, Doni A, Garlanda C, Mantovani A (2010). An integrated view of humoral innate immunity: pentraxins as a paradigm. Annu Rev Immunol.

[42] Sanchez-Mejias A, Tay Y (2015). Competing endogenous RNA networks: tying the essential knots for cancer biology and therapeutics. J Hematol Oncol.

[43] Shen J, Zhang J, Jiang X, Wang H, Pan G (2018). LncRNA HOX transcript antisense RNA accelerated kidney injury induced by urine-derived sepsis through the miR-22/high mobility group box 1 pathway. Life Sci.

[44] Chen Y, Qiu J, Chen B, Lin Y, Chen Y, Xie G, Qiu J, Tong H, Jiang D (2018). Long non-coding RNA NEAT1 plays an important role in sepsis-induced acute kidney injury by targeting miR-204 and modulating the NF-κB pathway. Int Immunopharmacol.

[45] Xu G, Mo L, Wu C, Shen X, Dong H, Yu L, Pan P, Pan K (2019). The miR-15a-5p-XIST-CUL3 regulatory axis is important for sepsis-induced acute kidney injury. Ren Fail.

[46] Ma P, Zhang C, Huo P, Li Y, Yang H. A novel role of the miR-152-3p/ERRFI1/STAT3 pathway modulates the apoptosis and inflammatory response after acute kidney injury. J Biochem Mol Toxicol. 2020. 10.1002/jbt.22540. 10.1002/jbt.22540 32583487

[47] Tan J, Fan J, He J, Zhao L, Tang H (2020). Knockdown of LncRNA DLX6-AS1 inhibits HK-2 cell pyroptosis via regulating miR-223-3p/NLRP3 pathway in lipopolysaccharide-induced acute kidney injury. J Bioenerg Biomembr.

